# The therapeutic potential of natural products for treating pancreatic cancer

**DOI:** 10.3389/fphar.2022.1051952

**Published:** 2022-11-02

**Authors:** Xia He, Ning Wang, Yu Zhang, Xiaobo Huang, Yi Wang

**Affiliations:** ^1^ Department of Pharmacy, Sichuan Academy of Medical Science and Sichuan Provincial People’s Hospital, University of Electronic Science and Technology of China, Chengdu, China; ^2^ Department of Critical Care Medicine, Sichuan Academy of Medical Science and Sichuan Provincial People’s Hospital, University of Electronic Science and Technology of China, Chengdu, China; ^3^ Department of Surgery, Sichuan Academy of Medical Science and Sichuan Provincial People’s Hospital, University of Electronic Science and Technology of China, Chengdu, China

**Keywords:** pancreatic cancer, natural products, curcumin, toosendanin, triptolide, panax notoginseng saponins

## Abstract

Pancreatic cancer is one of the most malignant tumors of the digestive tract, with the poor prognosis and low 5-year survival rate less than 10%. Although surgical resection and chemotherapy as gemcitabine (first-line treatment) has been applied to the pancreatic cancer patients, the overall survival rates of pancreatic cancer are quite low due to drug resistance. Therefore, it is of urgent need to develop alternative strategies for its treatment. In this review, we summarized the major herbal drugs and metabolites, including curcumin, triptolide, *Panax Notoginseng* Saponins and their metabolites *etc.* These compounds with antioxidant, anti-angiogenic and anti-metastatic activities can inhibit the progression and metastasis of pancreatic cancer. Expecting to provide comprehensive information of potential natural products, our review provides valuable information and strategies for pancreatic cancer treatment.

## Introduction

Pancreatic cancer (PC) is one of the leading causes of cancer-related deaths worldwide. It has been known for its difficult in diagnosis and poor prognosis even with surgery and chemotherapy. The symptom of PC is hard to be perceptible, therefore the majority of patients were found to be the late stage of disease. Moreover, these patients are inoperable due to the severe metastasis and invasiveness (Eiznhamer and Xu, 2004). Lots of factors contribute to the oncogenesis and progression of pancreatic cancer, including the environment and lifestyle, alcohol, high fat diet (Rawla et al., 2019), genetic mutation of onco-hub genes as *KRAS* and other oncogenes as *CDKN2A*, *TP53* and *SMAD4* (Dixon et al., 2012; Zhang et al., 2020), epigenetic regulation (Xiao et al., 2015; Yu et al., 2018; Yu et al., 2020; Yu et al., 2021), hyperglycemia (Chien K and Liao, 2016; Hu et al., 2019), and chronic pancreatitis (Kichler and Jang, 2020; Piersma et al., 2020).

Currently, surgery and adjuvant chemotherapy are considered to be the only viable options for pancreatic cancer treatment. However, 80% of patients are not suitable for surgery due to the invasiveness and metastasis of tumor cells. Some chemotherapeutic drugs and immunotherapy have been applied for these patients, but the pharmacological effects are limited (Hanahan and Weinberg, 2011; Holohan et al., 2013; Wang et al., 2022a). Hence, there is an urgent need for finding novel potential therapeutic drugs.

Traditional Chinese medicine has been applied in east asia for thousands of years and some of these natural botanical drugs, including paclitaxel, camptothecin and podophyllotoxin have successfully applied in clinics for years (Xu et al., 2011; Fedoros et al., 2018; Pan et al., 2020; Zhou et al., 2022a). Therefore, in light of summarizing the potential natural products for pancreatic cancer treatment, we comprehensively addressed the natural products and some major anti-cancer metabolites.

## Current therapeutic strategy of pancreatic cancer

Current treatment options for pancreatic cancer include surgery, immunotherapy and targeted therapy. Surgical resection is the major treatment for pancreatic cancer (Bockhorn et al., 2014). Immunotherapy and targeted therapies are currently new approaches to the treatment of pancreatic cancer. Immunotherapy includes tumor vaccine, adoptive cell immunotherapy, oncolytic virus therapy, immune checkpoint inhibitors, etc (Conroy et al., 2018). Targeted therapy mainly includes targeted therapy for tumor angiogenesis, targeted therapy of KRAS or other proteins of oncogenes and related signaling pathways. (Cao et al., 2021; Liu et al., 2021).

## Natural products undergoing clinical evaluation in pancreatic cancer

Natural products refer to the constituents or their metabolites in animals, plant extracts or insects, marine organisms and microorganisms, which are collectively referred to as natural products. For decades, natural products played an important role in the development of anti-tumor drugs, and many natural products such as paclitaxel, vincristine or their analogs have been widely used in clinical practice (Flores-Bustamante et al., 2010). These natural products exert their antitumor activities through different or novel mechanisms of action. Hence, we updated more information for botanical drugs other than paclitaxel and vincristine. Table 1 summarizes the therapeutic effects of some natural products on pancreatic cancer.

**TABLE 1:** Botanical drugs or metabolites for pancreatic cancer.

Category	Natural products	Source	Cell line/Animal model	Efficacy	Mechanism	Yes/No to enter clinical trials	References
Triterpenoids	Toosendanin	*Melia azedarach* L	PANC-1, AsPC-1 cells and BALB/c nude mice	Inhibits cell growth and migration, inhibits tumor production	Inhibit EMT transition, downregulation of Akt/mTOR signaling	No	Pei et al. (2017)
Diterpenoids	Triptolide	*Tripterygium wilfordii* Hook. f	MiaPaCa-2, Capan-1 and BxPC-3 cells	Induction of apoptosis	caspase-dependent apoptotic death, suppression of hedgehog signaling	No	(Ding et al., 2015; Qiao et al., 2016; Dai et al., 2019; Feng et al., 2019; Ma et al., 2019; Zhao et al., 2020)
	Libertellenone H	*Eutypellasp*.D-1	PANC-1, SW 1990, AsPC-1, BxPC-3, HPDE6-C7 cells	Induction of apoptosis	Induces ROS accumulation *via* Trx system, triggers autophagy	No	Zhang et al. (2021b)
Glycosides	Propolis	Bee	Panc-1 cells	Induction of apoptosis	Hippo-YAP signaling pathway	No	(Liu et al., 2018; He et al., 2019; Tao et al., 2021)
Saponin	Panax Notoginseng Saponins	*Panax notoginseng (Burkill) F. H. Chen ex C. H*	MiaPaCa-2, PANC-1, Panc‐1/GEM and SW1990/GEM cells	Inhibit proliferation, migration, invasion and autophagy	Caspase-dependent apoptosis, ZFP91 mediated TSPYL2 destabilization	No	(Tang et al., 2013; Guo et al., 2014; Jiang et al., 2017; Zou et al., 2020)
Flavonoids							
	Xanthohumol	*Humulus lupulus* Linn	PANC-1, PSN-1, MS1, BxPC-3 cells, AsPC-1 cells and MIA PaCa-2 cells	Induction of apoptosis	Regulates the activity of NF-κB and Nrf2	No	(Jiang et al., 2015; Kunnimalaiyaan et al., 2015; Saito et al., 2018)
	Curcumin	*Curcuma longa* L	BxPC-3, SW1990 cells, nuce mice, pancreatic cancer patients	Inhibit proliferation and promote apoptosis	IL-6/ERK/NF-κB axis	Yes (Phase II: NCT00094445)	(Malhotra et al., 2021; Chen et al., 2022a; Huang et al., 2022; Jie et al., 2022; Malhotra et al., 2022)
	Pterostilbene	*Pterocarpus indicus* Willd	MIA PaCa-2 cell	Inhibit proliferation and promote apoptosis	expression *via* the RAGE/PI3K/Akt axis	No	(Benlloch et al., 2016; Hsu et al., 2020; Chen et al., 2021a; Obrador et al., 2021)
Nucleosides	Cordycepin	*Cordyceps militaris* (L.) Link	BxPC-3, CFPAC-1, AsPC-1, PANC-1, SW 1990, MIAPaCa-2 and Capan-1 cells	Inhibit proliferation and promote apoptosis	caspase-dependent apoptosis, inhibit pro-inflammation cytokines, downregulate NF-κB) and NLRP3	No	(Zhang et al., 2018b; Li et al., 2020; Yang et al., 2020)

Abbreviations: EMT, Epithelial–mesenchymal transition; mTOR, mammalian target of rapamycin; ROS, reactive oxygen species; YAP, yes associated transcriptional regulator; Nrf2, Nuclear factor erythroid 2-related factor 2; IL-6, interleukine-6; ZFP91, Zinc finger protein 91 homolog; TSPYL2, Y-encoded-like 2; PI3K, Phosphatidylinositol 3-kinase; Akt, protein kinase B; ERK, Extracellular-signal-regulated kinase RAGE, receptor for advanced glycosylation end products; NF-κB, nuclear factor-κB; NLRP3, NLR, family pyrin domain-containing protein 3.

### Curcumin

Curcumin is a plant polyphenolic compound extracted from the *Curcuma longa* L. of the ginger plant turmeric. It is also known as diferuloylmethane, and was first isolated in 1815. Curcumin has a broad variety of pharmacological effects, including anti-inflammatory, antioxidant, anti-tumor, enhancing radiotherapy and chemotherapy sensitivity, and protecting liver and kidney functions (Hewlings and Kalman, 2017). It has been broadly investigated in cancers, including acute myeloid leukemia (Zhou et al., 2022b), prostate cancer (Al-Rabia et al., 2022), breast cancer, lung cancer etc (Giordano and Tommonaro, 2019).

For the pre-clinical study of curcumin, great progress has been made on both its mechanisms. In BxPC-3 cells, curcumin could restore mutant p53(Y220C) function and promote apoptosis in a dose- and time-dependent manner (ranging from 2 µM to 10 μM, 12–72 h, respectively) (Malhotra et al., 2021; Malhotra et al., 2022). Curcumin could synergically suppress the pancreatic cancer cell (SW1990 cell) proliferation with 10058-F4, an inhibitor of c-Myc both *in vitro* and *in vivo* (Jie et al., 2022). Curcumin analogue C66 has been reported to inhibit the proliferation and migration of pancreatic cancer in a dose- and time-dependent manner (25–100 μM, 24–72 h, respectively). Further this analogue could inhibit the inflammatory cytokines (IL-1β, IL-6, IL-8, and IL-15) secretion via the inhibition of JNK pathway (Chen et al., 2022a). The anti-pancreatic cancer effects and mechanisms of curcumin and its derivatives are described in detail (Huang et al., 2022).

Curcumin is the only metabolite or botanical drug under clinical study for pancreatic cancer. From 2004 to 2010, a total of 44 patients diagnosed of pancreatic neoplasms adenocarcinoma, were enrolled in a phase II clinical trial of curcumin. In collaboration with Sabinsa Corporation, this trial is performed at M.D. Anderson Cancer Center. Curcumin is delivered orally at the dosage of 8 g/day for 8 weeks. The median age included in this study is 65 (range: 40-87) with 56% female. Meanwhile, it is neither an open-label study nor randomized trial. The outcome of this trial is not disclosed by the researchers. We only know that nine of the 44 patients has serious adverse effects, including cardiac disorders (chest pain, multiple pulmonary emboli, *etc.*), gastrointestinal disorders (chronic cancer progression, metastasis, abdomen pain, *etc.*) and other disorders (Kim et al., 2021).

### Triptolide

Triptolide is an epoxy diterpene lactone compound extracted from the roots, leaves, flowers and fruits of *Tripterygium wilfordii Hook. f.* Triptolide has a broad range of pharmacological effects on autoimmune diseases, including rheumatoid arthritis (Li et al., 2022a), systemic lupus erythematosus (Zhang et al., 2022a), ankylosing spondylitis (Ji et al., 2022), and so on. Due to poor water solubility (0.017 mg/ml) and severe toxicity including excessive immune responses, its clinical application is greatly limited (Ding et al., 2017), and several strategies has been applied to reduce its toxicity and to improve its solubility (Kang et al., 2022a; Zhang et al., 2022b; Rao et al., 2022).

Triptolide has also been applied in a broad range of cancers. It could synergistically increase apoptosis of gastric cancer cells with Tumor necrosis factor-α (TNF-α) *via* the inhibition of H19/miR-204-5p/NF-κB/FLIP axis (Yuan et al., 2022). Followed by the decreased DNA (cytosine-5-)-methyltransferase 1 (DNMT1) and DNA (cytosine-5-)-methyltransferase 3a (DNMT3a) expression and the inhibition of Wnt inhibition factor 1 (WIF1), Sex-determining region Y-box (SOX17), cadherin 1 (CDH1) and Secreted Frizzled Related Protein 5 (SFRP5) demethylation, triptolide could inhibit the Wnt pathway, and thereby it inhibits T-cell acute lymphoblastic leukaemia (Ma et al., 2022). For the malignant melanoma treatment, targeted delivery of triptolide was applied with cyclopeptide and αvβ3 integrin-specific exosomes (Gu et al., 2022). Accumulating evidences revealed that triptolide has a strong antitumor effect in a broad range of cancers, including non-small cell lung cancer (Zhou et al., 2022c), colorectal cancer (Liskova et al., 2022; Song et al., 2022), hepatocellular carcinoma (Li et al., 2022b), acute myeloid leukemia (Chen et al., 2022b; Kang et al., 2022b) and so on.

For the treatment of pancreatic cancer, triptolide at the dose of 10–40 nM could inhibit pancreatic cancer cell proliferation through the suppression of hedgehog signaling pathway (Feng et al., 2019), or *via* the inhibition of plasminogen activator urokinase (PLAU) (Zhao et al., 2020). In another study, 50 nM triptolide could increase TRAIL sensitivity, downregulate the PUM1, and stimulate autophagy of pancreatic cancer cell (Dai et al., 2019). Study on BxPC-3 cells, PANC-1 cells and transplanted tumor models revealed that triptolide, at the dose of 0.1–1 µM could improve the gemcitabine sensitivity *via* the suppression of TLR4/NF-κB signaling pathway (Qiao et al., 2016; Ma et al., 2019). At the dose of 10–50 ng/ml, triptolide could also inhibits the proliferation by suppressing hypoxia-inducible factor-1α (HIF-1α) and c-Myc expression (Ding et al., 2015). It induces cell death by O-GlcNAc modification of transcription factor Sp1((Banerjee et al., 2013)). It can also be modified as prodrug. Chitosan oligosaccharide (CSO) conjugated triptolide could improve the water solubility to 15 mg/ml) (Wang et al., 2022b). Triptolide could also be conjugated with 2-(pyridin-2-yldisul-fanyl)ethyl acrylate (PDA)- poly (ethylene glycol) methyl (PEG) and lactobionic acid (LBA) (Sui et al., 2021). Three amino acids (tryptophan, valine, and lysine) based triptolide prodrug could also inhibit the growth of pancreatic cancer (Lou et al., 2021). In combined with a cytoprotective agent, diammonium glycyrrhizinate (DG), a complex lipid emulsion (TP/DG-CLE) could increase the therapeutic effects of triptolide (Mu et al., 2022).

### *Panax Notoginseng* Saponins and their metabolites

*Panax Notoginseng* Saponins (PNS) is the main chemical component of the *Panax notoginseng* (Burkill) F. H. Chen ex C. H. PNS has multiple pharmacological functions, including the inhibition of the platelet aggregation, improving microcirculation, inhibiting the inflammation responses and reducing the oxidative stress (Zhang et al., 2018a).

Recent research on PNS revealed its anti-cancer activity and low toxicity in various cancers, including prostate cancer, colorectal cancer, retinoblastoma, and so on (Han et al., 2018; Li et al., 2022c; Hawthorne et al., 2022; Zhong et al., 2022). Study on pancreatic cancer Miapaca2 and PANC-1 cells revealed that the IC_50_ of PNS are 377.1 and 492.5 µM, respectively, whiles gemcitabine is the positive control. PNS could limit the proliferation, migration, invasion of pancreatic cell proliferation, and induce the autophagy of these cells. Importantly, it could stimulate the apoptosis and chemosentivity to gemcitabine *via* caspase-dependent pathway (Yao et al., 2021). Gold nanoparticles from the leaf of PNS has been proved to possess anti-pancreatic cancer activity in PANC-1 cells (Wang et al., 2019).

Further studies on the metabolites of PNS revealed that there are ginsengosides have anti-proliferative function on pancreatic cells. Haixia Pan et al. found that 50–200 µM ginsenoside Rg3 could increase the chemosensitivity of gemcitabine on pancreatic adenocarcinoma through the inhibition of Zinc finger protein 91 homolog (ZFP91) mediated testis specific Y-encoded-like protein 2 (TSPYL2) destabilization (Pan et al., 2022). Another study on ginsenoside Rg3 demonstrated that same dose of ginsenoside Rg3 could suppresses the growth of gemcitabine-resistant pancreatic cancer cells (Panc‐1/GEM and SW1990/GEM cells). Molecular mechanism investigation revealed that ginsenoside Rg3 could upregulate lncRNA-CASC2 and activates PTEN signaling pathway (Zou et al., 2020). In BxPC-3 and AsPC-1 cells, it stimulates the apoptosis and increase the anti-proliferative effects of erlotinib *via* the decreased phosphorylation of EGFR, PI3K, and Akt (Jiang et al., 2017). It inhibits the angiogenesis of pancreatic cancer via the downregulation of VE-cadherin/EphA2/MMP9/MMP2 signaling pathway (Guo et al., 2014). Furthermore, 20–60 µM ginsengoside Rh2 could also induce Bxpc-3 cell cycle arrest, and reduced migration and invasion in a caspase dependent manner (Tang et al., 2013).

### Toosendanin

Toosendanin is a tetracyclic triterpenoid extracted from the fruit or bark of the plant *Melia toosendan Sieb. et Zucc*. or *Melia azedarach L.* Toosendanin is a white crystalline powder, easily soluble in methanol, ethanol and pyridine (Shi and Li, 2007). With an IC50 of 26 nM, toosendanin could inhibit the growth of a broad range of tumors. By inhibiting the Hh-involved Hedgehog pathway, toosendanin coud inhibit colorectal cancer cell growth (Zhang et al., 2022c). As an autophagy inhibitor, it could block autophagy by inhibiting the activity of vacuolar-type H^+^-translocating ATPase, induce necroptosis and promote apoptosis in triple-negative breast cancer cells and tumor xenograft models (Dong et al., 2022; Zhang et al., 2022d; Zhang et al., 2022e). Through the inhibition of the PI3K/Akt/mTOR signaling pathways, toosendanin could inhibit the growth of glioma cells and tumor growth *in vivo* animal models (Zhang et al., 2021a). In the hepatocellular carcinoma, toosendanin functions as WW-domain containing oxidoreductase (WWOX) agonist and thereby inhibits tumor metastasis by the inhibition of JAK2/Stat3 and Wnt/β-catenin signaling pathways (Yang et al., 2021a; Yang et al., 2021b). It could induce the apoptosis of ovarian cancer and gastric cancer in a caspase-dependent pathway and through the κ-opioid receptor/β-catenin signaling axis (Shao et al., 2020; Wang et al., 2020; Wang et al., 2021a).

Pancreatic cancer cells were treated with different concentrations of toosendanin (50, 100 and 200 nM, respectively), and the results showed that pancreatic cancer cell lines had apoptosis in a dose dependent manner (Pei et al., 2017). Besides, toosendanin can also inhibit the migration and invasion of pancreatic cancer cells at the dosage of 200 μM.

Further mechanism investigation revealed that toosendanin could reverse the TGF-β induced epithelial-mesenchymal transition through increasing the expression of Ecadherin and reducing the expression of Vimentin, ZEB1 and SNAIL. It also suppressed TGF-β mediated EMT *via* the deactivation of Akt/mTOR signaling pathway. In the *in vivo* xenograft mice experiments, toosendanin at a dose of 0.2 mg/kg. Taken together, toosendanin shows an activity that can specifically inhibit pancreatic cancer cells (Pei et al., 2017).

### Xanthohumol and phenethyl isothiocyanate

Xanthohumol, a flavonoid isolated from *Humulus lupulus* L., has anti-inflammatory, anti-tumor and anti-angiogenesis properties (Krajka-Kuźniak et al., 2013; Niederau et al., 2022; Vesaghhamedani et al., 2022). It could reduce cell viability and cause G2/M arrest at the dose of 40 μM through MAPK JNK pathway for human nasopharyngeal carcinoma cells (Hsieh et al., 2022). At 10 nM dose, it could inhibit the colon cancer cellproliferation and progression *via* downregulating inflammatory signals (TNF-α and IL-6) and glucose metabolism (Torrens-Mas et al., 2022). By increasing p53-upregulated modulator of apoptosis (PUMA)-mediated apoptosis, it also inhibits non-small cell lung cancer (Li et al., 2022d). It hampers glutamine uptake in triple negative breast cancer (Carmo et al., 2022). It also inhibits a variety of cancers as prostate, B-chronic lymphocytic leukemia, hepatocellular, and medullary thyroid cancers (Lust et al., 2005; Szliszka et al., 2009; Cook et al., 2010; Dorn et al., 2010).

Xanthohumol could inhibit the growth of MiaPaCa-2, PANC-1, AsPC-1, and L3.6 pl cells at a dose-dependent manner, ranging from 5 mM to 30 mM. It can also inhibit PC patient-derived cells in a dose-dependent manner as well. Further mechanisms studies revealed that xanthohumol mediated cell apoptosis *via* the inhibition of Notch 1, HES-1 and survivin at both transcription and translation levels (Kunnimalaiyaan et al., 2015). It could also induce cell cycle arrest of PANC-1 and BxPC-3 cells by inactivating signal transducer and activator of transcription 3 (STAT3) and downstream genes as cyclinD1, survivin, and Bcl-xL (98). Xanthohumol has been reported to suppress angiogenesis by inactivation of NF-κB, and inhibiting the vascular endothelial growth factor (VEGF) and interleukin-8 (IL-8) expression both *in vitro* (BxPC-3 cells, AsPC-1 cells and MIA PaCa-2 cells, 10–25 µM) and *in vivo* (10 mg/kg) (Saito et al., 2018). Xanthohumol, in combination with phenethyl isothiocyanate (extracted from *Oenanthe javanica* (Bl.) DC.) could inhibit the PSN-1 cell growth with an IC_50_ 46 µM and inhibit the MS1 cell growth with an IC_50_ 49 µM. This combination could greatly reduce the expression of NF-κB p65 subunits, and activate Nrf2 and downstream genes as GSTP, NQO1, and SOD in PANC-1 cells (Krajka-Kuźniak et al., 2020) and PSN-1 cells (Cykowiak et al., 2021). Further investigation revealed that the combination of xanthohumol (40 mg/kg) and phenethyl isothiocyanate (15 mg/kg) could inhibit the plasma COX-2 and nuclear STAT3 expression (Cykowiak et al., 2021).

### Pterostilbene

Pterostilbene (3,5-dimethoxy-40 -hydroxystilbene) is a phytoalexin, the secondary metabolite isolated from the heartwood of red sandalwood *pTocarpus santalinus*. Pterostilbene has been reported to be an efficient anti-cancer agents in hepatocellular carcinoma with IC_50_ of about 20–40 μM in various HCC cell lines. Meanwhile, it has similar inhibitory function in drug (sorafenib and lamivudine)-resistant HCC cell through the inhibition of ribonucleotide reductase activity, and thereby inhibit virus replication (Qian et al., 2018; Wang et al., 2021b).

Studies on pancreatic ductal adenocarcinoma (PDAC) demonstrated that 10–75 µM pterostilbene could enhance chemosensitivity by the inhibition of MIA PaCa-2 cell proliferation and cell cycle arrest. It could also induce both apoptosis and autophagy. Further mechanic study revealed that pterostilbene inhibited multidrug resistance protein 1 (MDR1) expression through the Receptor for advanced glycosylation end products (RAGE)/PI3K/Akt signaling pathway (Hsu et al., 2020). It can also increase cell death by inducing lysosomal membrane permeabilization (Obrador et al., 2021). Chloroquine could substantiate the anti-tumor effects of pterostilbene on PC through the inhibition of autophagy *via* downregulating RAGE/STAT3 and protein kinase B (AKT)/mammalian target of rapamycin (mTOR) signaling pathway (Chen et al., 2021a). *In vivo* study using AsPC-1 and BxPC-3 cells xenograft mice revealed that 20–40 mg/kg pterostilbene downregulates the glucocorticoid secretion *via* reducing glucocorticoid receptor and inhibiting Nrf2-dependent signaling pathway (Benlloch et al., 2016).

### Cordycepin

Cordyceps militaris is the mycelium of *Cordyceps militaris* (L.) Link. It has the functions of anti-tumor and immune regulation. Cordycepin is one of its main active components, which has the activity of regulating immunity, antibacterial and anti-inflammatory (Pan et al., 2015; Kong et al., 2022). It has been broadly investigated in various cancers, including triple-negative breast cancer (Wei et al., 2022; Wu et al., 2022), lymphoma (Shi et al., 2022), colon cancer (Deng et al., 2022), glioblastoma (Chen et al., 2022c) and so on both *in vitro* and *in vivo*. It could also increase the radiosensitivity by cell cycle arrest, ER stress and caspase dependent apoptosis (Lee et al., 2022). It could induce autophagy in tumor cells (Chen et al., 2021b).

Cordycepin has been reported to induce the caspase-dependent apoptosis in a dose dependent manner. 50–100 µM cordycepin suppress the growth pancreatic cancer cell growth with an IC_50_ of 38.85, 72.99, 150.1, 213.1, and 349.3 μM for BxPC-3, CFPAC-1, AsPC-1, PANC-1 and SW1990 cells, respectively. It induces S-phase arrest *via* activating checkpoint kinase 2 (Chk2) and downregulating cyclin A2 and CDK2 phosphorylation, inhibits Ras/ERK pathway (Li et al., 2020). In human pancreatic cancer cells (MIAPaCa-2 and Capan-1 cells), cordycepin could induce apoptosis in a dose- and time-dependent manner *in vitro* (ranging 50–600 μM, 24–72 h, respectively) and *in vivo* (15and 50 mg/kg/d for 28 days) *via* mitochondrial mediated intrinsic apoptosis pathways (Zhang et al., 2018b). Furthermore, for acute pancreatitis therapy, cordycepin could also be potential treatment by inhibiting the pro-inflammatory cytokines as IL-6, IL-1β, and TNF-α *via* the inhibition of nuclear factor-κB (NF-κB) and NLR family pyrin domain-containing protein 3 (NLRP3) (Yang et al., 2020).

### Libertellenone H

Libertellenone H (LH) is a marine-derived diterpene-type diterpenoid isolated from the high latitude lived arctic fungus *Eutypella* sp. D-1 (120). In the anti-tumor assay, the positive controls are adriamycin, 5-fluorouracil and paclitaxel, and LH has been proved to inhibit the growth of U251 cells, SW-1990 cells, SG7901 cells, MCF-7 cells, Huh-7 cells, Hela cells, and H460 cells (Lu et al., 2014).

For pancreatic cancer treatment, it could inhibit the growth of human pancreatic cancer cell lines PANC-1 (IC_50_, 3.21 μM), SW 1990 (IC_50_, 0.67 μM), AsPC-1 (IC_50_, 2.78 μM), BxPC-3 (IC_50_, 5.53 μM) and human pancreatic duct epithelial cells HPDE6-C7 (IC_50_, 10.86 μM) in a dose-dependent manner (Zhang et al., 2021b). Further investigation revealed that it could stimulate ROS accumulation *via* the suppression on the Trx and the corresponding receptor system. Covalently binds toTrx1 and TrxR, it activates ASK1/JNK signaling pathway (Zhang et al., 2021b).

### Other herbal drugs or metabolites

Mangiferin, 1,3,6,7-tetrahydroxyxanthone-C2-β-D-glucoside, is extracted from the leaves and barks of mango tree (Mangifera indica L.). It possesses a variety of pharmacological activities, such as antioxidative, antitumor, antibacterial, antiviral, anti-diabetes, and so on (Wang et al., 2014; Du et al., 2018; Wang et al., 2018). It could inhibit the proliferation of lung cancer (Shi et al., 2016), ovarian cancer (Zou et al., 2017; He et al., 2019), breast cancer, glioblastoma (Mu et al., 2018) and other cancers (Du et al., 2018). Recent studies on mangiferin revealed that it could inhibit the Mia-PaCa2 cell proliferation in a dose-dependent manner ranging from 3.06 to 100 µM. Further mechanic study indicated that 5, 10, and 20 µM mangiferin could induce autophagy, and caspase-dependent apoptosis. Additionally, it could induce the G2/M arrest and endogenous ROS production (Yu et al., 2019).

Propolis is a natural product obtained by mixing resin collected by bees and their own secretions. The chemical composition of propolis is very complex, which is affected by various factors such as its plant source, the type of bees collected and the collection time. In recent years, many studies have proved that propolis from Mexico, Brazil, Vietnam and other places has an inhibitory effect on the proliferation of pancreatic cancer cells (Awale et al., 2008; Li et al., 2010; Nguyen et al., 2017). For example, the Algerian bee limb can enhance the anti-pancreatic cancer effect mediated by doxorubicin by regulating apoptosis, cell cycle and inhibiting the activity of P-glycoprotein (Rouibah et al., 2018). The active ingredient CAPE in propolis can induce apoptosis of pancreatic cancer Panc-1 cells through mitochondrial dysfunction and activation of caspase-3/-7 (Chen et al., 2008). In addition, Chinese propolis inhibits the proliferation of human pancreatic cancer Panc-1 cells through the Hippo-YAP signaling pathway (Liu et al., 2018; He et al., 2019). The IC_50_ value of Propolis on human pancreatic cancer Panc-1 cell is about 50 μg/ml. Treatment with 50μg/ml Propolis for 48 h resulted in 34.25 ± 3.81% apoptosis of human pancreatic cancer Panc-1 cells. After treatment with propolis, the expression of YAP in human pancreatic cancer Panc-1 cells was significantly reduced, and its nuclear entry was inhibited. At the same time, the expression levels of the main upstream proteins MST1 and LATS1 and the downstream phosphorylated effector protein p-YAP were significantly increased. These studies will justify the research and development of pharmaceuticals and related health products containing propolis (Tao et al., 2021).

## Conclusion and perspectives

Accumulating evidences revealed that metabolites or botanical drugs could efficiently inhibits pancreatic cell proliferation and induces apoptosis or autophagy *in vitro* and alleviates the progression of cancer in xenograft mouse models. Because botanical drugs have been used in the world for thousands of years, especially in east asia, the advantages and adverse effects have been tested known. However, most studies of these botanical drugs are in the primitive stage. Data from *in vitro* cell study and *in vivo* xeograft mouse research are not enough for pre-clinical study. More animal studies as rabbit, beagle and non-human primate studies are needed for the new drug development of these botanical drugs. Meanwhile, these studies will cover the gap from bench to bed. Among these studies, curcumin is under phase II clinical trial of pancreatic neoplasms adenocarcinoma sponsored by researchers from M.D. Anderson Cancer Center. However, the detailed outcome of this trial is not released by the researcher and we could only find pieces of information about the adverse effects of curcumin, including cancer progression and metastasis. As most of these metabolites/botanical drugs are still based on *in vitro* cell study and *in vivo* rodent data, the major bottleneck for the clinical trial is no large animal study is available. How to shorten the gap between pre-clinical research and clinical trial is still challenges for researchers and clinicians. And more non-human primate studies should be performed for the efficacy and safety of these metabolites/botanical drugs before clinical trials. With more and more in-depth investigations on large animals, metabolites or botanical drugs will ultimately be applied in clinics in the near future.
